# Global Health in the medical curriculum

**DOI:** 10.6061/clinics/2021/e3073

**Published:** 2021-05-27

**Authors:** Nelson Gouveia, José Ricardo de Carvalho Mesquita Ayres

**Affiliations:** Departamento de Medicina Preventiva, Faculdade de Medicina FMUSP, Universidade de Sao Paulo, Sao Paulo, SP, BR.

In recent decades, the term “Global Health” has appeared with increasing frequency in academic debates, technical documents from the health field, and scientific literature. Related concepts such as “health diplomacy,” “global public health,” “global health governance,” and “planetary health” have also been considerably prominent (1). Therefore, it is not surprising that many medical schools are restructuring their curricula to include content related to this “new” area of knowledge, which has resulted in the emergence of Global Health as a new disciplinary field (2).

In fact, the notion of Global Health has been gradually replacing International Health--the well-known inductive policy promoted in large part by the Rockefeller Foundation's work with medical schools in developed countries since the beginning of the last century, which mainly focused on the control of so-called tropical diseases. The interventions developed were mainly aimed at the control of epidemics across borders and at actions adopted by low- and middle-income countries for the prevention and control of communicable diseases and those related to sanitation, malnutrition, and maternal and child health (3,4). Criticisms on the restricted scope, the characteristic assistentialism, and the largely colonialist stance of old International Health practices contributed to the emergence of Global Health. However, many academic departments and organizations continue to use the previous name despite reorienting to the new tendencies, whereas others have adhered to the new name without significantly transforming their approaches (5).

Global Health came about from the perception that several major health problems are affected by factors resulting from globalization, such as the increased mobility of people, products, resources, and lifestyles and the economic interdependence between countries--conditions that have led to a new level of interconnectivity and the increasing international transfer of risks and opportunities (3,4,6). Globalization has intensified the notion that no nation or organization is capable of independently dealing with the health threats it faces. The emergence and re-emergence of infectious diseases, especially the recent influenza, Ebola, dengue, and Zika epidemics and the current COVID-19 pandemic; the increased mobility of immigrants and refugees, which results in greater ethnic and cultural diversity of populations and more complex challenges to epidemiological surveillance; and the growing burden of diseases related to urbanization, environmental pollution, climate change, and a model of economic development that has deepened the differences between the rich and the poor, generating poverty, violence, and illness are examples of health issues whose understanding and coping demand “geographically broad and historically profound” approaches (7).

There are various definitions of Global Health. However, all of them emphasize that health issues, determinants, and solutions are transnational phenomena that transcend geographical boundaries and are better addressed by cooperative actions and solutions that involve multidisciplinary and interdisciplinary collaboration both within and beyond the health sciences (4,8,9). Global Health should not be considered to be restricted only to issues that literally cross borders, but should include any problem--local, regional, or global--that is determined or affected by transnational practices or conditions. The “global” in Global Health refers to the scope of the problems, not just their location (4).

The field of action of Global Health has imprecise limits and interfaces with multiple disciplines or areas of knowledge. Law is one of those particularly important interface areas, not only because many health problems require the establishment of agreements and international regulations for their solution, but also because the relations between health and the promotion, protection, and respect for human rights are striking (10). The field of International Relations, which examines Global Health governance, that is, the management of actions or coordinated responses to certain problems, also has an important interface with Global Health (6). Finally, it is also worth remembering Global Health Ethics--a relatively new field that has been used to conceptualize the process of applying a moral value to health issues that are characterized by an effect at the global level or that require coordinated action at the global level (11).

Given its growing importance, interest in teaching Global Health has been increasing among medical professors and students and new skills related to this field have been incorporated into health education in general, and medical training in particular (12,13). The curricula of several schools contain themes such as understanding the global burden of diseases and the main causes of morbidity and mortality and its variations between populations, the social and environmental determinants of health, equity in health and social justice, the different models of national health systems, the World Health Organization’s system of surveillance of infectious diseases and their articulation within our local systems with emphasis on the International Health Regulations and the Public Health Emergency of International Concern, traveler’s medicine, the health of refugee, migrant, or transitory populations, health care disparities between countries, primary care within diverse cultural contexts, and skills to better interact with different populations, cultures, and health systems, and especially the link between health and human rights, among other diverse skills (12-14).

The integration of these and other topics in the context and practice of clinical medicine does not necessarily require the creation of new disciplines. For example, this integration can occur through the identification of relevant existing content that is presently dispersed within the curriculum, which can be subsequently refined to suit the contemporary need. It can also be done by integrating such content within existing disciplines. As for postgraduate studies, more specific and specialized disciplines with greater theoretical and methodological depth can be planned in order to train researchers, teachers, and professionals in leadership and innovation in the field of Global Health.

Given that health care in the contemporary milieu involves the aforementioned complex issues, the skills provided by the field of knowledge and practices of “Global Health” become necessary for a health professional to undertake a more active role. This expertise enables them to understand local, national, and international scenarios and their interconnections and to use cultural and cross-cultural skills that meet global health needs. As already indicated by Fortes and Ribeiro (3), Global Health is based on the notion of supra-territoriality, but establishes connections from the global to the local level.

In an increasingly globalized and connected world, it is important to invest in the training of professionals who can transform the realities of local and global health by working at the different levels of health care complexity in order to promote, protect, recover, and rehabilitate the health of the population effectively and equitably.

## AUTHOR CONTRIBUTIONS

Gouveia N and Ayres JRCM contributed to the manuscript drafting and review, and were also responsible for the approval of the final version submitted.
